# Mesenchymal stromal cells ameliorate systemic sclerosis-interstitial lung disease via PD-1/PD-L1 signalling axis

**DOI:** 10.1136/rmdopen-2025-006324

**Published:** 2026-01-06

**Authors:** Yuxuan Chen, Huimin Zhu, Yue Zhang, Mian Liu, Yingyi Wu, Shuai Ding, Dandan Wang, Lingyun Sun

**Affiliations:** 1 Department of Rheumatology and Immunology, Nanjing Drum Tower Hospital, Affiliated Hospital of Medical School, Nanjing University, Nanjing, Jiangsu, China; 2 Department of Rheumatology and Immunology, Nanjing Drum Tower Hospital, Clinical College of Jiangsu University, Nanjing, Jiangsu, China; 3 Department of Rheumatology and Immunology, Nanjing Drum Tower Hospital Clinical College of Nanjing University of Chinese Medicine, Nanjing, Jiangsu, China; 4 Department of Rheumatology and Immunology, China Pharmaceutical University Nanjing Drum Tower Hospital, Nanjing, Jiangsu, China

**Keywords:** Scleroderma, Systemic, Immune System Diseases, Pulmonary Fibrosis

## Abstract

**Objective:**

Systemic sclerosis-associated interstitial lung disease (SSc-ILD) is characterised by progressive pulmonary fibrosis. This study aimed to investigate the role of programmed death-1 (PD-1)-expressing T cells in SSc-ILD pathogenesis and evaluate the therapeutic potential and mechanism of mesenchymal stromal cells (MSCs) in mitigating fibrosis.

**Methods:**

PD-1 expression in T cells from 30 patients with SSc (including SSc-ILD and SSc-non-ILD (nILD) subgroups) and 15 healthy controls (HCs) was analysed via flow cytometry. A bleomycin (BLM)-induced SSc-ILD mouse model was established to evaluate the effects of MSCs in the treatment of lung collagen deposition and inflammation in SSc-ILD. MSCs were administered intravenously to BLM-treated mice, with programmed death-ligand 1 (PD-L1) knockdown (using small interfering RNA targeting PD-L1, siPD-L1) used to explore the mechanism of MSCs on PD-1/PD-L1 pathway. The effects of MSCs on CD4^+^PD-1^+^ T cell proliferation and apoptosis were evaluated by in vitro co-culture experiment.

**Results:**

PD-1 expression was significantly elevated in CD3^+^ and CD4^+^ T cells of patients with SSc-ILD compared with HCs and SSc-nILD subgroups. In BLM-induced mice, CD4^+^PD-1^+^ T cells in the lung tissues increased progressively, which was correlated with the severity of lung fibrosis. CD4^+^PD-1^+^ T cells directly stimulated fibroblasts to upregulate the expression of collagen and transforming growth factor β1. Treatment with MSCs reduced pulmonary inflammation, fibrosis and PD-1^+^ T cell frequencies in lung tissues of BLM-induced mice. This therapeutic effect was PD-L1-dependent, as it was mediated by the MSC-induced suppression.

**Conclusion:**

CD4^+^PD-1^+^ T cells drive fibrosis in SSc-ILD, and MSCs ameliorate disease by suppressing PD-1^+^ T cells through PD-L1-mediated mechanisms. These findings highlight PD-1 as a therapeutic target and support the clinical investigation of MSC-based interventions for SSc-ILD.

**WHAT IS ALREADY KNOWN ON THIS TOPIC**

- Programmed death-1 (PD-1) upregulation in T cells has been implicated in autoimmune diseases, and mesenchymal stromal cells (MSCs) exhibit immunomodulatory properties.
- Previous studies suggest a potential role of PD-1/programmed death-ligand 1 (PD-L1) signalling in fibrotic processes, but its specific contribution to systemic sclerosis-associated interstitial lung disease (SSc-ILD) remains unclear.

**WHAT THIS STUDY ADDS**

- CD4^+^PD-1^+^ T cells identified as key drivers of pulmonary fibrosis in SSc-ILD.
- MSCs ameliorate fibrosis by suppressing PD-1^+^ T cells via PD-L1-dependent mechanisms.

**HOW THIS STUDY MIGHT AFFECT RESEARCH, PRACTICE OR POLICY**

- PD-1 as a therapeutic target in SSc-ILD and provide preclinical evidence supporting MSC-based therapies.

## Introduction

 A rare autoimmune condition, systemic sclerosis (SSc) is defined by a complex pathology involving immune system dysregulation. Its clinical hallmarks include the thickening of cutaneous tissue, progressive fibrosis affecting various internal organs and widespread damage to the vasculature.^1^ This disease predominantly affects women and manifests as either limited cutaneous SSc or diffuse cutaneous SSc, with the latter subtype exhibiting a particularly aggressive progression and an elevated risk of developing interstitial lung disease (ILD).^2^ Among the various complications associated with SSc, ILD is one of the most prevalent and severe, significantly impairing lung function and diminishing the quality of life for affected individuals.^3^ Pulmonary fibrosis occurs in up to 80% of patients with SSc.^4^ The pathogenesis of SSc-ILD is complex, involving abnormal functions in multiple cellular and molecular pathways. In the early stage, SSc-ILD is characterised by progressive pulmonary fibrosis, vascular disease and inflammatory infiltration, especially lymphocyte and macrophage infiltration.^5^,^7^ The management of SSc-ILD is complex due to the heterogeneity of the disease and the limited number of approved treatment options. Current therapeutic strategies primarily focus on immunosuppressive therapies and emerging antifibrotic agents. Nevertheless, these pharmacological agents have demonstrated limited efficacy in randomised clinical trials, as they are unable to reverse established fibrosis. Additionally, adverse reactions have impacted their tolerability, and there remains a necessity for further validation regarding their long-term efficacy and safety.^8^,^10^ These unmet clinical needs underscore the urgency of new treatment strategies.

Mesenchymal stromal cells (MSCs) have emerged as a promising candidate due to their dual antifibrotic and immunomodulatory capacities.^11 12^ In our previous clinical trial, after umbilical cord-derived MSCs (UC-MSCs) transplantation, patients with moderate-to-severe SSc had improved 5-year and 10-year survival rates. For those with SSc-ILD, high-resolution CT (HRCT) showed stable or improved pulmonary fibrosis.^13 14^ In addition, Fang *et al* developed multifunctional engineered MSCs to promote angiogenesis and reshape the vascular structure of the fibrotic region, thereby promoting lung tissue regeneration and reversing pulmonary fibrosis.^15^ Animal studies indicate that bone marrow-derived MSCs can reduce skin and lung fibrosis, lower IgG1/IgG2a antibody levels in lung tissue and decrease airway hyper-responsiveness.^16^ Additionally, other murine studies have shown that MSCs can alleviate SSc-associated inflammation and fibrosis by inducing T cell apoptosis via the Fas/FasL pathway, promoting the generation of regulatory T cells (Tregs) and decreasing fibroblast activation and proliferation.^17^ However, the precise mechanisms remain elusive.

Emerging evidence suggests programmed death-1 (PD-1) checkpoint dysregulation may play pivotal roles in SSc pathogenesis. PD-1 is upregulated on CD4^+^ T cells in patients with SSc, particularly within the PD-1^high^ CXCR5^−^HLA^–^DR^+^ICOS^−^ subset, which is associated with disease activity and severity of ILD.^18^ This CD4^+^ T cell population exhibits increased granzyme expression and is linked to microvascular injury and tissue fibrosis. CD4^+^PD-1^+^ T cells, especially PD-1^high^CXCR5^−^ cells, promote fibrosis through interleukin (IL)-17A and transforming growth factor (TGF)-β1 production, regulated via signal transducer and activator of transcription 3 (STAT3) signalling.^19^ In vitro and animal studies show that PD-1 pathway blockade reduces collagen-1 production by fibroblasts and attenuates bleomycin (BLM)-induced lung fibrosis in mice.^20^ These collective findings position PD-1 in pathogenic T cell subsets as a key orchestrator, establishing PD-1 blockade as a potential therapeutic target to disrupt the immune-fibrotic axis in SSc-associated pulmonary fibrosis.

Our study integrates clinical observations with mechanistic validation in animal models. Building on previous reports indicating that human MSCs upregulate programmed death-ligand 1 (PD-L1) under inflammatory conditions,^21^ we hypothesise that MSC therapy ameliorates SSc-ILD by reprogramming the PD-1/PD-L1 axis in CD4^+^ T cell subsets. This study not only analyses how MSC-driven modulation of PD-1 reshapes the immune-fibrotic cascade but also advances precision MSC therapy by identifying the PD-1/PD-L1 axis as a modifiable target to enhance treatment efficacy.

## Materials and methods

### Patients

Between October 2022 and August 2023, 30 patients with SSc and 15 healthy volunteers were recruited from the Rheumatology and Immunology Department at the Affiliated Drum Tower Hospital, Nanjing University Medical School. All patients with SSc were diagnosed based on the 2013 American College of Rheumatology (ACR)/European Alliance of Associations for Rheumatology (EULAR) new Classification Criteria for Systemic Sclerosis.^22^ Patients were classified into SSc-ILD and SSc-non-ILD (nILD) according to HRCT, lung function and clinical status.^23^ The detailed clinical data and CT scores are presented in online supplemental table 1. The CT scoring was based on a systematic HRCT assessment where the overall burden of abnormalities (including ground-glass opacity, consolidation, traction bronchiectasis, reticulation, honeycombing and emphysema) in each lung was graded using a 4-point system. For this assessment, chest HRCT was conducted with 1.0–1.5 mm thick sections and viewed with optimised window parameters (width: 1600, level: −600).

### Mice models

C57BL/6J female mice were housed in a specific pathogen free (SPF) facility. The animals, aged 7–8 weeks and weighing 20–25 g, were procured from GemPharmatech. The BLM group received a continuous intratracheal infusion of BLM (2.5 U/kg) for 21 days, while the control group was given phosphate buffer saline (PBS). All animal experiments were approved by the Committee of Experimental Animal Administration of the Affiliated Drum Tower Hospital of Nanjing University Medical School (project ID: 20200722).

### Cell sorting of T cells

The isolation of CD4^+^ T cells from peripheral blood mononuclear cells (PBMCs) was performed in accordance with the supplier’s guidelines (Miltenyi Biotec, Bergisch Gladbach, Germany). For the subsequent identification of a specific subset, these purified cells were incubated with a BV421-conjugated antibody against mouse PD-1. The PD-1 expressing cells were then isolated by fluorescence-activated cell sorting on a BD FACSAria system.

### MSCs cell culture and PD-L1 knockdown

MSCs were cultured in DMEM/F12 medium (Biochannel, Nanjing, China) supplemented with 10% fetal bovine serum (FBS; ExCell, China) and 1% penicillin-streptomycin (Invitrogen, New York, New York, USA). The cultures were maintained in an incubator at 37°C with 5% CO_2_ and were passaged through passages 6–7. *PD-L1* knockdown of MSCs (KeyGEN Biotech, Nanjing, China) was performed using human-targeted small interfering RNA against PD-L1 (siPD-L1, siPD-L1 #1: GCCGAAGUCAUCUGGACAATT, siPD-L1 #2: GAAGCAAAGUGAUACACAUTT, siPD-L1 #3: GCAGUGACCAUCAAGUCCUTT). At 24 hours post-transfection, cells were collected for subsequent evaluation and experiments.

### MSCs treatment

To verify the efficacy of UC-MSCs in the treatment of SSc-ILD pulmonary fibrosis, female C57B/6J mice were divided into three groups (PBS, BLM and BLM+MSCs, n=4 per group) and received 5×10^5^ MSCs or the same volume of PBS via tail vein on days 3 and 7 post-BLM administration. Mice were sacrificed 2 weeks post-treatment for further analysis. To verify the effect of MSCs on PD-1, female C57B/6J mice were divided into four groups (PBS, BLM, BLM+MSCs and BLM+MSCsiPD-L1, n=4 per group), the purified MSCs and PD-L1-knockdowned MSCs (5×10^5^ cells/mouse) were injected into mice intravenously on day 3 and day 7 after BLM treatment. Mice were sacrificed 2 weeks post-treatment for further analysis.

Patients with SSc-ILD are considered non-responders or to be significantly dissatisfied with conventional immunosuppressive therapy if the following conditions are present: (1) A lack of therapeutic response despite over 6 months of treatment with immunosuppressants, including continuous or concurrent use of cyclophosphamide (CTX, 500–750 mg/m^2^/month) or mycophenolate mofetil (MMF, >1000 mg/day). (2) A worsening of skin fibrosis, indicated by an increase of 20% or more in the modified Rodnan skin score (mRSS). (3) The development of significant organ involvement attributable to SSc.^13^ The treatment protocol consisted of a single intravenous infusion of UC-MSCs at a dose of 1×10^6^ cells/kg of body weight. The day of MSC infusion was defined as day 0, and the day before infusion was day −1, serving as the baseline.

### Proliferation and apoptosis assay

CD4^+^ T cells were purified from PBMCs as directed by the manufacturer (Miltenyi Biotec, Bergisch Gladbach, Germany). For proliferation analysis, the cells were stained with 5 µM carboxyfluorescein diacetate succinimidyl ester (CFSE, Invitrogen) in PBS at 37°C for 10 min, a reaction stopped with excess cold 10% FBS-supplemented RPMI 1640 medium. These labelled T cells (1×10^6^/well) were then co-cultured for 3 days with MSCs (1:10 ratio) in the presence of 1 µg/mL anti-CD3/CD28 stimulation. Proliferation was measured as the dilution of CFSE fluorescence by flow cytometry.

For apoptosis detection, the cultured CD4^+^ T cells were resuspended in 100 µL of 1×binding buffer (10 mM N-2-Hydroxyethylpiperazine-N-2-Ethane Sulfonic Acid (HEPES), 140 mM NaCl, 2.5 mM CaCl₂) and labelled with 5 µL of Fluorescein isothiocyanate (FITC)-annexin V (BD Biosciences) for 15 min in the dark at room temperature. The frequency of apoptotic cells was analysed immediately using a flow cytometer.

### Flow cytometry analysis

To isolate cells, lung tissues were first mechanically dissociated using scissors and then enzymatically processed with 1 mg/mL collagenase II for 30 min. The enzymatic reaction was neutralised by adding PBS. The resulting cell mixture was passed through a cell strainer to remove debris, followed by centrifugation at 1500 rpm for 5 min. After lysing red blood cells, a single-cell suspension was obtained.

Cell viability and concentration were determined using a haemocytometer. For staining, the cell count was normalised to 1×10^6^ cells per sample. For the analysis of murine lung infiltrates, samples were stained with a cocktail of antibodies from BioLegend, including Zombie Aqua (BV510) for viability, anti-mouse PD-1 (BV421), anti-mouse CD3 (allophycocyanin (APC)/Cy7) and anti-mouse CD4 (BB700).

For the characterisation of human peripheral blood T cells, a separate antibody panel from eBioscience was used, consisting of anti-human CD3 (FITC), anti-human CD4 (APC) and anti-human PD-1 (BV786).

### Histological evaluation of lung tissues

For histological analysis, lungs were first perfused in situ with cold saline to remove blood before excision. The right lung lobes were immediately snap-frozen in liquid nitrogen for later molecular analysis. The left lung lobes were preserved by fixation in a 10% formalin buffer solution.

These fixed tissues were subsequently embedded in paraffin blocks and sectioned into 4 µm serial slices. Prior to staining, the sections underwent a complete deparaffinisation and rehydration process. To assess the extent of pulmonary inflammation and fibrosis, H&E staining and Masson staining were conducted according to the manufacturers’ standard protocols. The Ashcroft score was assessed according to the previous method. Ashcroft scores were assigned to evaluate the extent of pulmonary fibrosis in lung sections stained with H&E and Masson.^24^

### Immunohistochemistry

Paraffin-embedded mouse lung tissue sections (4 µm) were deparaffinised, rehydrated and subjected to heat-induced antigen retrieval in citrate buffer (pH 6.0). After blocking endogenous peroxidase and non-specific binding, the sections were incubated overnight at 4°C with primary antibodies against α-smooth muscle actin (α-SMA, Proteintech, #14395–1-AP, 1:4000), CD3 (Proteintech, #17617–1-AP, 1:1000), CD4 (Abcam, #ab183685, 1:1000) or CD8 (Abcam, #ab217344, 1:2000). Staining was visualised using an horseradish peroxidase (HRP)-conjugated secondary antibody and a 3,3' -diaminobenzidine (DAB) substrate kit. Sections were then counterstained with haematoxylin, dehydrated and mounted. Images were acquired using a standard light microscope.

### Real-time PCR

For gene expression analysis, total RNA was extracted from mouse lung tissues and the L929 cell line using Trizol reagent. A reverse transcriptase kit (Vazyme, Nanjing, China) was employed to reverse-transcribe the RNA into complementary DNA (cDNA). Quantitative real-time PCR was then performed using a 2×SYBR Green Master Mix (Vazyme). The 10 µL reaction volume included 1 µL of cDNA template and 10 µmol/L of forward and reverse primers. Gene expression was quantified using the comparative cycle threshold (Ct) method, with β-actin serving as the internal reference for normalisation. The manufacturer’s recommended protocol was followed for all amplification steps.

### Hydroxyproline content assay

The degree of pulmonary fibrosis was assessed by quantifying the hydroxyproline (HYP) content in lung tissues using a commercial kit (Solarbio, Cat. No. BC0255). Briefly, weighed lung tissues were hydrolysed at 100°C for 4 hours. After centrifugation, the supernatant was incubated with chromogenic reagents at 60°C for 20 min. The absorbance was measured at 560 nm. The HYP concentration was determined by comparison with a standard curve, and the results were normalised to the wet tissue weight (μg/g). All procedures were performed according to the manufacturer’s protocol.

### Statistical analysis

All statistical computations were carried out using GraphPad Prism software (V.8.0.2; San Diego, California, USA). Numerical results are expressed as the mean±SD. Comparisons between groups were performed using either a one-way analysis of variance or an unpaired Student’s t-test, as appropriate for the experimental design. A p value was calculated to determine statistical significance.

## Results

### CD4^+^PD-1^+^ T cells upregulated in SSc-ILD

**Figure 1:**
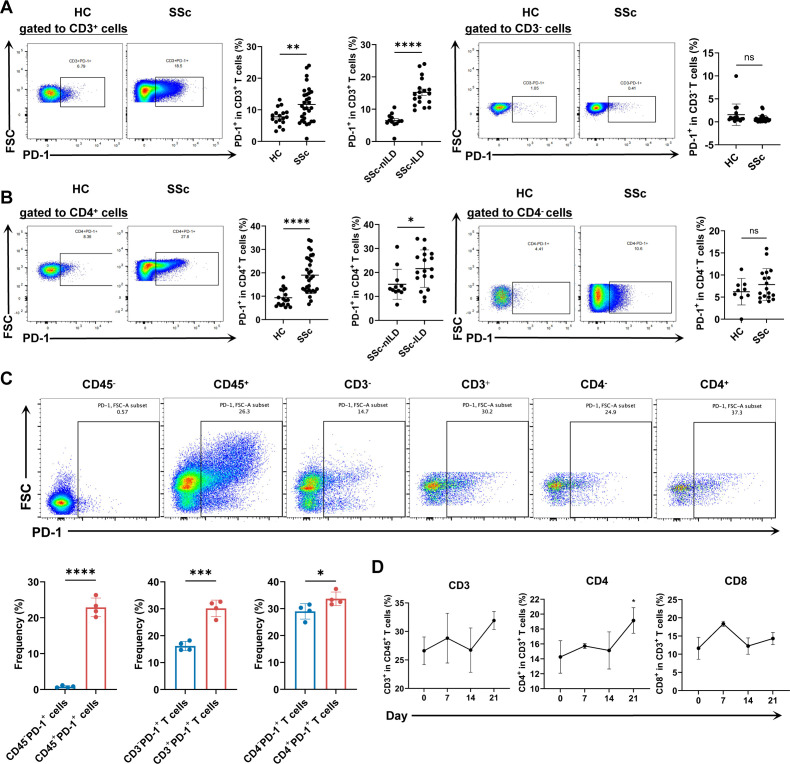
Analysis of immune cell phenotypes in patients with SSc and the BLM-induced SSc-ILD model. (**A**) Frequencies of PD-1^+^ cells within peripheral blood CD3^+^ and CD3^−^ populations from patients with SSc (n=30) and HCs (n=15). (**B**) Frequencies of PD-1^+^ cells within peripheral blood CD4^+^ and CD4^−^ T cell populations from patients with SSc (n=30) and HCs (n=15). (**C**) Frequencies of PD-1^+^ cells among different pulmonary lymphocyte subsets in BLM-induced mice (n=4 per group). (**D**) Frequencies of CD3^+^, CD4^+^ and CD8^+^ T cells among CD45^+^ cells at days 0, 7, 14 and 21 after BLM instillation in C57BL/6J mice (n=4 per timepoint). All data are expressed as means±SD. P value was calculated using one-way ANOVA and unpaired t-test. *p<0.05, **p<0.01, ***p<0.001, ****p<0.0001. ANOVA, analysis of variance; BLM, bleomycin; FSC, forward scatter; HC, healthy control; ILD, interstitial lung disease; nILD, non-ILD; ns, not significant; PD-1, programmed death-1; SSc, systemic sclerosis.

The expression of PD-1 in T cell subsets from the blood of 30 patients with SSc and 15 healthy controls (HCs) was analysed by flow cytometry. The results showed that CD3^+^ T cells demonstrated higher PD-1 expression than their CD3^−^ counterparts, with a similar pattern observed in CD4^+^ T cells. Patients with SSc had increased percentages of PD-1^+^ cells among CD3^+^ T cells compared with HCs, and patients with SSc-ILD showed higher percentages than patients with SSc-nILD. When examining specific T cell types, the proportion of PD-1^+^ cells within the CD4^+^ T cell population was significantly greater in patients with SSc than in HCs, and in patients with SSc-ILD compared with patients with SSc-nILD. In contrast, no significant differences were found in CD4− T cells between any groups (figure 1A,B). To explore these findings further, we established a BLM-induced SSc-ILD mouse model. In these mice, PD-1 was primarily found on lung CD45^+^ immune cells rather than on other cell types, with higher expression on CD3^+^ T cells compared with non-T cells and highest levels on CD4^+^ T cells among all T cells (figure 1C). Longitudinal analysis revealed that CD4^+^ T cells progressively increased from day 7 to day 21, while CD8^+^ T cells only temporarily rose at day 7 before declining (figure 1D). Taken together, these results demonstrate that PD-1-expressing CD4^+^ T cells continuously expand during SSc-ILD development and are closely associated with disease progression. This suggests that their important role in driving this condition is significant.

### CD4^+^PD-1^+^ T cells promote pulmonary fibrosis

**Figure 2:**
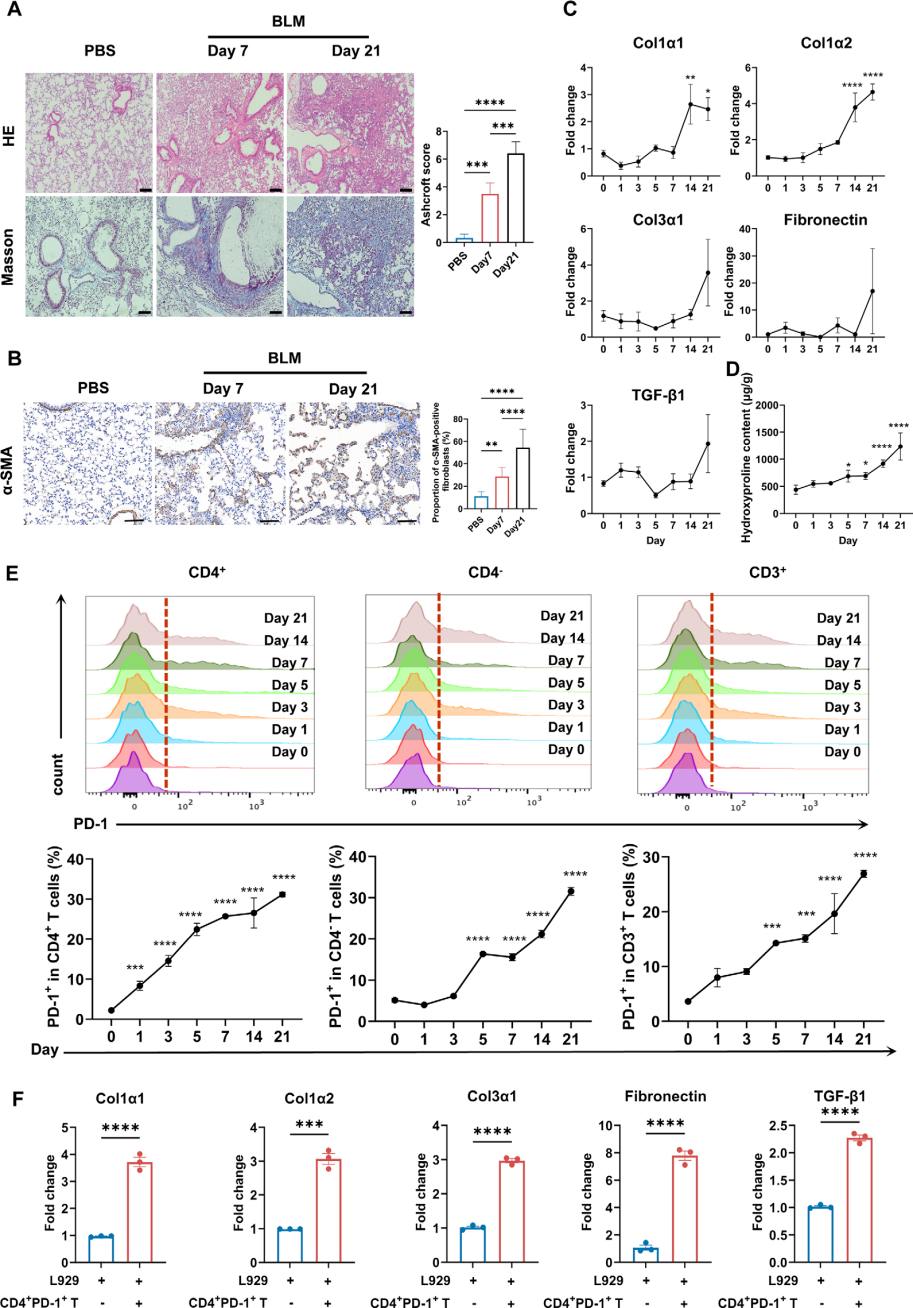
CD4^+^PD-1^+^ T cells promote fibrotic progression in the BLM-induced SSc-ILD model. (**A**) Representative images of H&E and Masson staining and corresponding Ashcroft fibrosis scores of lung sections from control mice (PBS) and from BLM-treated mice at 7 and 21 days post-injection. Scale bar=100 µm. (**B**) Representative α-SMA immunohistochemical staining of lung sections from control mice and from BLM-treated mice at 7 and 21 days post-injection. Scale bar=100 µm. (**C**) Relative mRNA expression levels of *Col1α1*, *Col1α2*, *Col3α1*, *fibronectin* and *Tgf-β1* in lung tissues on days 0, 1, 3, 5, 7, 14 and 21 after the BLM instillation in C57BL/6J mice (n=4). (**D**) Hydroxyproline content in lung tissues at the indicated time points after BLM instillation (n=4 per timepoint). (**E**) Frequencies of PD-1^+^ cells within CD3^+^, CD4^+^ and CD4^−^ T cell subsets in the lungs of C57BL/6J mice at indicated timepoints after BLM instillation (n=4 per timepoint). (**F**) Relative mRNA expression of fibrotic markers in L929 following co-culture with in vitro-isolated CD4^+^PD-1^+^ T cells (n=3). All data are expressed as means±SD. P value was calculated using one-way ANOVA. *p<0.05, **p<0.01, ***p<0.001, ****p<0.0001. ANOVA, analysis of variance; BLM, bleomycin; Col1α1, collagen type I alpha 1; Col1α2, collagen type I alpha 2; Col3α1, collagen type III alpha 1; ILD, interstitial lung disease; mRNA, messenger RNA; PBS, phosphate buffer saline; PD-1, programmed death-1; SSc, systemic sclerosis; TGF, transforming growth factor; α-SMA, α-smooth muscle actin.

To further explore how CD4^+^PD-1^+^ T cells contribute to pulmonary fibrosis, we examined inflammatory cell infiltration and collagen deposition in mouse lungs at different time points after BLM induction. BLM-treated lungs showed increased inflammatory cell infiltration and collagen deposition, peaking at day 21 (figure 2A,D). Similarly, pulmonary fibrosis, characterised by extensive collagen deposition and an increased fibroblast proportion, also reached its peak at day 21 (figure 2B,C). Critically, the proportion of PD-1-expressing cells within the CD4^+^ T cell population rose significantly starting on day 1 after BLM and continued increasing until day 21, while the proportion of PD-1^+^ cells among CD4^−^ T cells only increased from day 5 and plateaued between days 5 and 7. The overall proportion of PD-1^+^ cells within total CD3^+^ T cells followed a similar pattern to the CD4^−^ subset (figure 2E). To assess the direct profibrotic effect of CD4^+^PD-1^+^ T cells, we co-cultured them with the mouse fibroblast cell line L929 at a 2:1 ratio, with stimulation by soluble anti-CD3 and anti-CD28 antibodies. After 3–4 days, L929 fibroblasts exhibited upregulated messenger RNA (mRNA) expression of key fibrosis markers, including collagen type I alpha 1 (Col1α1), collagen type I alpha 2 (Col1α2), collagen type III alpha 1 (Col3α1), Fibronectin (Fn1) and transforming growth factor β1 (Tgf-β1) (figure 2F). These results demonstrate that CD4^+^PD-1^+^ T cells can directly drive the development of a profibrotic phenotype.

### MSCs treatment mitigates lung fibrosis of SSc-ILD model

**Figure 3:**
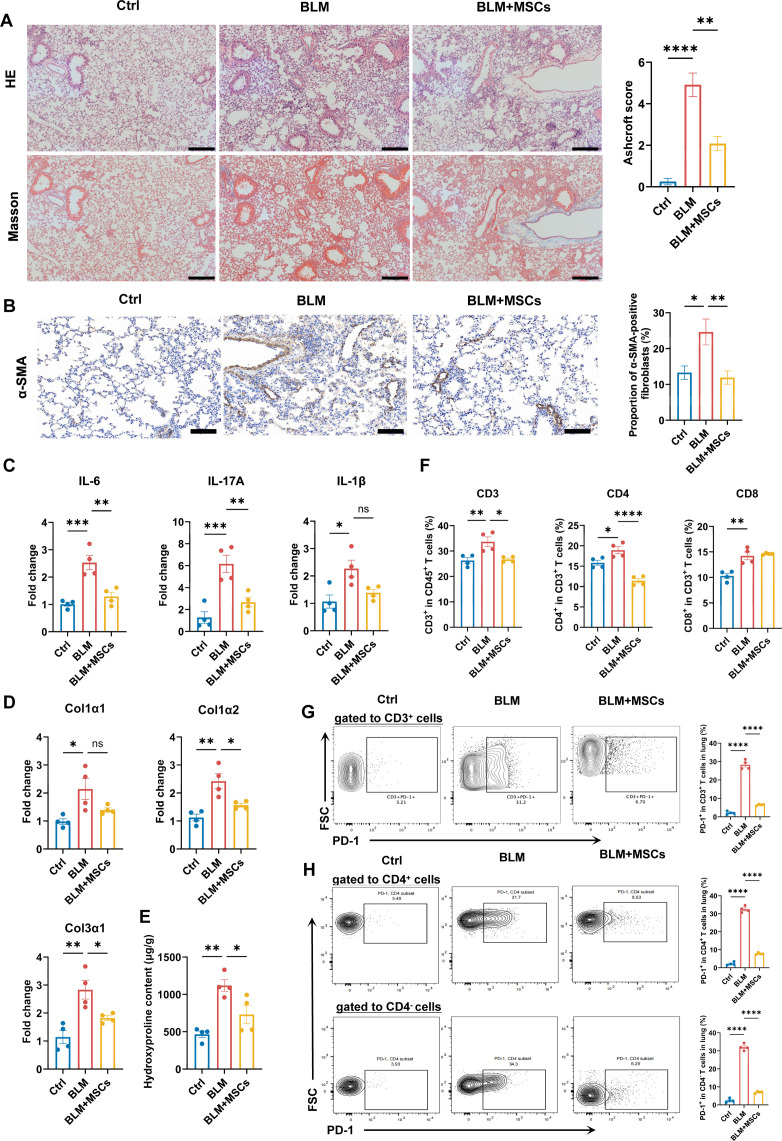
MSCs ameliorate inflammation and fibrosis and modulate T cell subsets in the BLM-induced pulmonary fibrosis model. (**A**) Representative images of H&E and Masson staining and corresponding Ashcroft fibrosis scores of lung sections. Scale bar=200 µm (n=4 per group). (**B**) Representative images of α-SMA immunohistochemical staining of lung sections (n=4 per group). Scale bar=100 µm. (**C**) Relative mRNA expression of inflammatory genes (*IL-6*, *IL-17A*, *IL-1β*) in the lung tissues of the three groups of mice. (**D**) Relative mRNA expression of fibrotic genes (*Col1α1*, *Col1α2*, *Col3α1*) in the lung tissues of the three groups of mice. (**E**) Hydroxyproline content in lung tissues, as a measure of collagen deposition. (**F**) Frequencies of CD3^+^, CD4^+^ and CD8^+^ T cells among CD45^+^ cells. (**G**) Frequencies of PD-1^+^ cells within the CD3^+^ T cell population. (**H**) Frequencies of PD-1^+^ cells within CD4^+^ and CD4^−^ T cell subsets. All data are expressed as means±SD. P value was calculated using one-way ANOVA. *p<0.05, **p<0.01, ***p<0.001, ****p<0.0001. ANOVA, analysis of variance; BLM, bleomycin; Col1α1, collagen type I alpha 1; Col1α2, collagen type I alpha 2; Col3α1, collagen type III alpha 1; Ctrl, control; FSC, forward scatter; IL, interleukin; mRNA, messenger RNA; MSCs, mesenchymal stromal cells; PD-1, programmed death-1; α-SMA, α-smooth muscle actin.

To investigate the therapeutic efficacy of MSCs on SSc-ILD in vivo, MSCs were infused into mice via the tail vein on day 3 and day 7, respectively, after BLM instillation. When compared with BLM group, the BLM+MSCs group showed significant amelioration of BLM-induced lung injury, evidenced by reduced inflammatory infiltration and collagen deposition (figure 3A, C and E). The degree of fibrosis was also attenuated, as indicated by a reduced number of fibroblasts (α-SMA^+^ cells) in the lung tissue (figure 3B). Furthermore, the mRNA expression of fibrotic mediators in lung tissues of BLM-treated mice was all diminished by MSC treatment (figure 3D). MSC treatment also reduced the elevated frequencies of CD3^+^ and CD4^+^ T cells in the lungs of the SSc-ILD model mice (figure 3F and online supplemental figure 1A). In the BLM-induced model, approximately 30% of pulmonary CD3^+^ T cells exhibited PD-1 expression. MSC treatment effectively attenuated the BLM-induced CD3^+^PD-1^+^ T cell frequency in lung tissues (figure 3G). Importantly, this therapeutic intervention significantly reduced the frequencies of PD-1^+^ cells within both the CD4^+^ and CD4^−^ T cell subsets (figure 3H).

### MSCs ameliorate SSc-ILD through the PD-1/PD-L1 pathway

**Figure 4:**
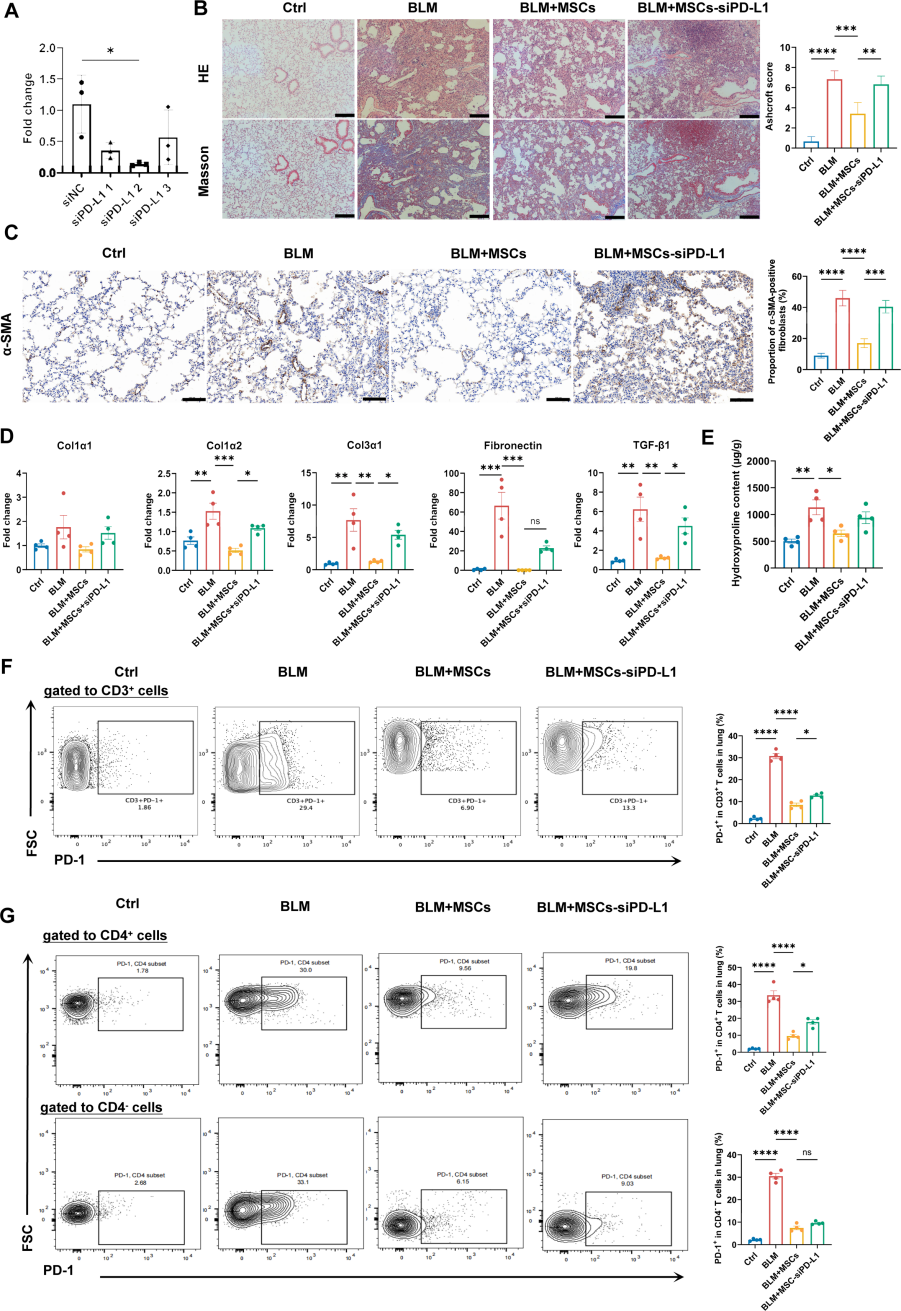
The therapeutic effect of MSCs is dependent on PD-L1 expression. (**A**) Validation of PD-L1 knockdown in MSCs by qRT-PCR following siPD-L1 transfection. (**B**) Representative images of H&E and Masson staining of lung sections and corresponding Ashcroft fibrosis scores. Scale bar=200 µm. (**C**) Representative images of α-SMA immunohistochemical staining of lung sections. Scale bar=100 µm. (**D**) Relative mRNA expression of key fibrotic and inflammatory genes in lung tissues. (**E**) The content of hydroxyproline in lung tissue. (**F**) Frequencies of PD-1^+^ cells within the pulmonary CD3^+^ T cell population. (**G**) Frequencies of PD-1^+^ cells within pulmonary CD4^+^ and CD4^−^ T cell subsets. All data are expressed as means±SD. P value was calculated using one way ANOVA. *p<0.05, **p<0.01, ***p<0.001, ****p<0.0001. ANOVA, analysis of variance; BLM, bleomycin; Col1α1, collagen type I alpha 1; Col1α2, collagen type I alpha 2; Col3α1, collagen type III alpha 1; Ctrl, control; FSC, forward scatter; mRNA, messenger RNA; MSCs, mesenchymal stromal cells; PD-1, programmed death-1; PD-L1, programmed death-ligand 1; qRT-PCR, quantitative real-time PCR; si, small interfering RNA; TGF, transforming growth factor; α-SMA, α-smooth muscle actin.

Previous studies have shown that PD-L1 expression on human MSCs is crucial for their immunomodulatory capacity in vitro, by targeting PD-1-expressing T cells.^25^ Therefore, we investigated whether the therapeutic effects of MSCs in our pulmonary fibrosis model were dependent on the PD-1/PD-L1 pathway. To test this, we knocked down *PD-L1* in MSCs using siPD-L1 prior to their administration (figure 4A). Importantly, the therapeutic effects of MSCs were largely abrogated on *PD-L1* knockdown. Specifically, treatment with siPD-L1-transfected MSCs failed to ameliorate pulmonary pathological damage (figure 4B,C), reduce the expression of profibrotic mediators (*Col1a2*, *Col3a1*, *Fn1*, *Tgf-β1*), or decrease hydroxyproline levels in the lungs (figure 4D,E). Furthermore, these PD-L1-deficient MSCs were unable to reduce the elevated frequencies of CD3^+^, CD4^+^ and CD8^+^ T cells in the lungs (online supplemental figure 1B). From these observations, we conclude that a pathway involving PD-L1 is a key contributor to the immunomodulation exerted by MSCs. This same mechanism was also implicated in reducing the severity of pulmonary fibrosis in our SSc-ILD model. In addition, the flow cytometry analysis revealed that MSC treatment significantly reduced the frequency of CD3^+^PD-1^+^ T cells in lung tissues, an effect that was reversed on PD-L1 knockdown in MSCs (figure 4F). Similarly, the inhibitory effect of MSCs on CD4^+^PD-1^+^ T cell frequency in lung tissues was abolished by PD-L1 knockdown. Notably, while MSCs also reduced the frequency of CD4^−^PD-1^+^ T cells, this alteration remained unaffected by PD-L1 knockdown in MSCs (figure 4G).

### MSCs inhibit the proliferation of CD4^+^PD-1^+^ T cells from patients with SSc-ILD

**Figure 5:**
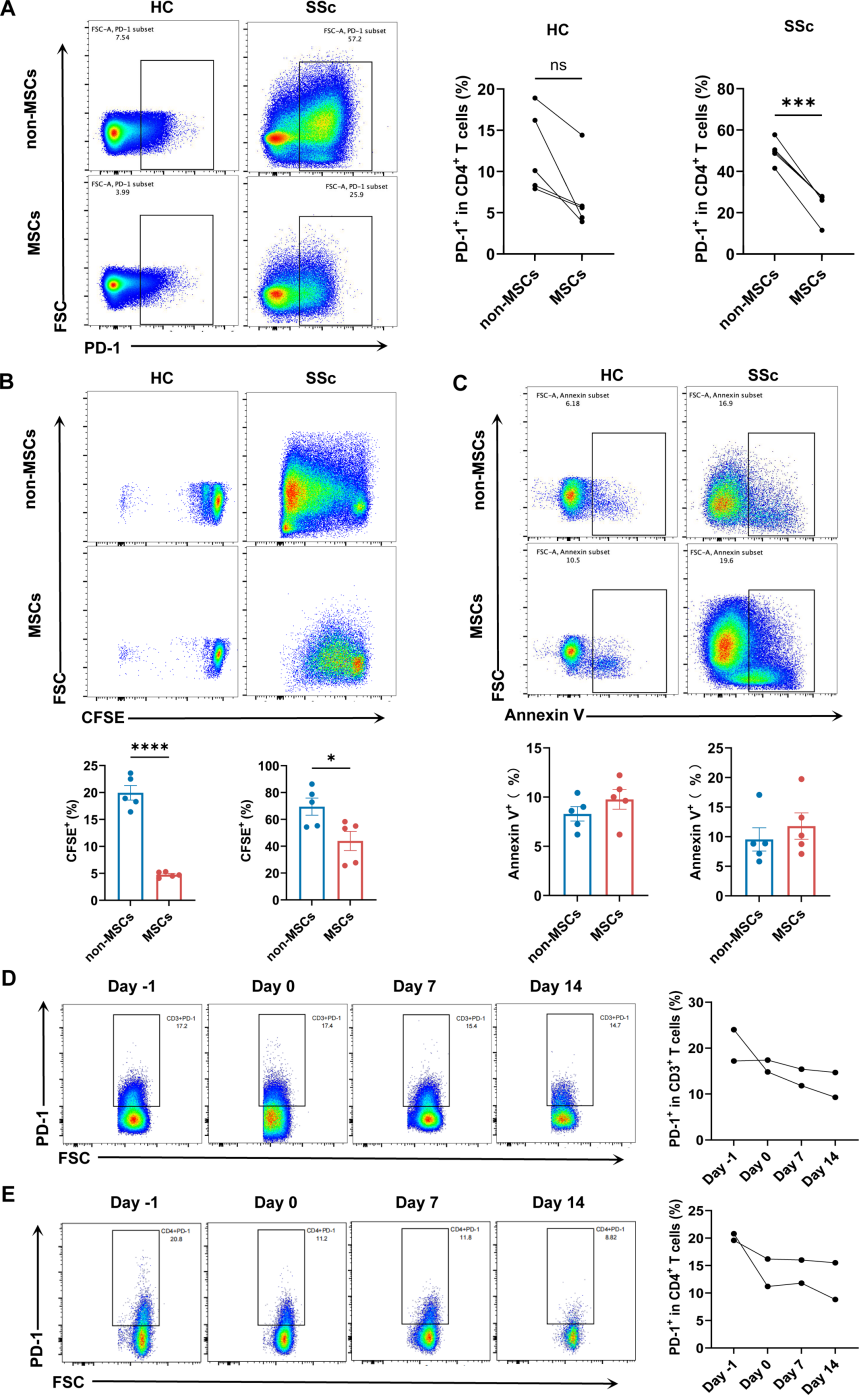
MSCs inhibit the proliferation, but not apoptosis, of CD4^+^PD-1^+^ T cells from patients with SSc-ILD. (**A**) Frequencies of PD-1^+^ cells within the CD4^+^ T cell population after a 3 day co-culture with or without MSCs. CD4^+^ T cells were isolated from patients with SSc-ILD or HCs (n=5 per group). (**B**) Apoptosis rates of CD4^+^PD-1^+^ T cells following co-culture of CD4^+^ T cells with or without MSCs (n=5 per group). (**C**) Proliferation of CFSE-labelled CD4^+^ T cells, measured as the frequency of divided CD4^+^PD-1^+^ T cells, after co-culture with or without MSCs (n=5 per group). (**D**) In vitro isolated PBMCs from patients with SSc-ILD treated with MSCs transplantation on days −1, 0, 7, 14 and the percentages of CD3^+^PD-1^+^ T cells in PBMCs were detected by flow cytometry. (**E**) In vitro isolated PBMCs from patients with SSc-ILD treated with MSCs transplantation on days −1, 0, 7, 14 and the percentages of CD4^+^PD-1^+^ T cells in PBMCs were detected by flow cytometry. All data were expressed as means±SD. P value was calculated using unpaired t test and one way ANOVA. *p<0.05, ***p<0.001, ****p<0.0001. ANOVA, analysis of variance; CFSE, carboxyfluorescein diacetate succinimidyl ester; FSC, forward scatter; HC, healthy control; ILD, interstitial lung disease; MSCs, mesenchymal stromal cells; ns, not significant; PBMCs, peripheral blood mononuclear cells; PD-1, programmed death-1; SSc, systemic sclerosis.

We investigated the immunomodulatory role of MSCs by co-culturing them with CD4^+^ T cells from patients with SSc-ILD and HCs. The results demonstrated that MSCs lowered the proportion of CD4^+^PD-1^+^ T cells in the patient group but not in the healthy group (figure 5A). To understand this inhibitory effect, we explored its impact on apoptosis and proliferation. While MSCs did not significantly alter the apoptosis of CD4^+^PD-1^+^ T cells in either cohort (figure 5B), they markedly attenuated the proliferation of these cells in both patients with SSc-ILD and HCs, as measured by a CFSE assay (figure 5C). Consistent with these in vitro findings, flow cytometric analysis of PBMCs from SSc patients after MSC therapy showed a significant decrease in the frequencies of both CD3^+^PD-1^+^ and CD4^+^PD-1^+^ T cells (figure 5D,E).

## Discussion

Our research presents that dysregulation of PD-1 in CD4^+^ T cells is integral to the pathogenesis of SSc-ILD and highlights the modulation of the PD-1/PD-L1 axis by MSCs as a crucial therapeutic mechanism. Three principal findings are elucidated: first, CD4^+^PD-1^+^ T cells are significantly enriched in both patients with SSc-ILD and corresponding murine models, which is correlated with the progression of fibrosis. Second, MSCs demonstrate dual anti-inflammatory and antifibrotic effects by inhibiting the proliferation of CD4^+^PD-1^+^ T cells. Third, the knockdown of PD-L1 negates the therapeutic efficacy of MSCs, thereby establishing this pathway as essential for the resolution of fibrosis.

SSc is a complex autoimmune disease characterised by multiorgan fibrosis, in which pulmonary fibrosis is one of the leading causes of death in patients. T cells are considered to be one of the key immune cells in the pathological process of SSc. The pathogenesis of SSc involves diverse T cell subset abnormalities, including Th17/Treg imbalance, Treg dysfunction, a decreased proportion of CD4^+^LAG3^+^ T cells, enhanced Th2 polarisation and so on. These aberrant T cell populations contribute to disease progression by secreting proinflammatory and profibrotic cytokines that stimulate collagen production in fibroblasts, while simultaneously impairing antifibrotic capabilities and disrupting the regulation of other immune cells within the body.^26^,^30^ In our study, the pivotal role of T cells in pulmonary fibrosis is further substantiated by histopathological correlations. Previous studies have also reported lymphocytic infiltration density in SSc-ILD lung tissues directly associates with disease severity, particularly in early inflammatory phase.^31^ Harada *et al* have reported that analyses of bronchoalveolar lavage fluid (BALF) fluid revealed a predominance of CD4^+^ T cells in fibrotic lungs. Furthermore, in idiopathic pulmonary fibrosis cases, elevated CD4/CD8 ratios in BALF correlate with accelerated functional decline,^32 33^ suggesting mechanisms across fibrotic lung diseases. In our study, in the BLM-induced mouse model of pulmonary fibrosis, the dynamism of the CD4^+^ T cell population was observed to progress in parallel with interstitial collagen deposition. This association suggests that CD4^+^ T cells not only participate in inflammatory responses but may also continuously influence the fibrotic process, likely through specific subsets.

PD-1 functions as a crucial immune checkpoint receptor, with its extracellular domain binding to the ligands PD-L1 and PD-L2. This interaction plays a significant role in modulating immune responses, particularly in T cells. Our research corroborates and extends these findings by demonstrating an increase in CD4^+^PD-1^+^ T cells both in patients with SSc-ILD and a mouse model of BLM-induced pulmonary fibrosis. Furthermore, these elevated levels are significantly correlated with the severity of interstitial lung disease.^18^ This means that PD-1 could play a crucial role in SSc-associated pulmonary fibrosis. We further substantiated this hypothesis in vitro by directly inducing fibroblast differentiation using CD4^+^PD-1^+^ T cells, likely through TGF-β1 and IL-17A overproduction, as demonstrated in our observations of BLM-induced pulmonary fibrosis in murine models.^19^ In other pulmonary fibrosis-related diseases, PD-1 also plays a key role in the fibrosis process. In silicosis patients, blocking of the PD-1/PD-L1 pathway can significantly reduce the degree of fibrosis, suggesting that this pathway plays an important role in the pathophysiological process of SSc-related pulmonary fibrosis.^34^

We further validated the efficacy of MSCs in the management of SSc-ILD. Preclinical studies demonstrated that MSC administration markedly attenuated lung fibrosis by reducing collagen deposition, restoring extracellular matrix homeostasis via elevated matrix metalloproteinase (MMP)-1/tissue inhibitor of metalloproteinase (TIMP)-1 and MMP-9/TIMP-1 ratios and suppressing profibrotic mediators such as TGF-β and platelet derived growth factor (PDGF) in BLM-induced, hypochlorous acid (HOCI)-induced and topoisomerase I (TOPO I)-induced models.^35 36^ Clinical studies further support the therapeutic potential of MSCs: a case–control study involving 113 patients with SSc treated with MSCs showed a significant improvement in long-term survival rates.^13^ In a 5-year follow-up study, 68.3% of patients with SSc with comorbid ILD exhibited improved pulmonary imaging findings, alongside enhanced quality of life and survival rates. Additionally, a case report highlighted that a patient with SSc-ILD experienced significant alleviation of clinical symptoms and radiological abnormalities after treatment with MSC-derived extracellular vesicles.^37^ Our in vivo experiments also confirmed that MSC application suppressed T-cell infiltration in BLM-induced lung tissues and was associated with a marked reduction in pulmonary fibrosis, further underscoring the immunomodulatory potential of MSCs in treating autoimmune-related lung fibrosis.

Previous studies have established that MSCs regulate fibroinflammatory responses through paracrine mechanisms in SSc-associated pulmonary fibrosis,^38^,^41^ but direct evidence for immune checkpoint-mediated regulation remained limited. This study demonstrated that MSCs primarily function through the PD-L1/PD-1 axis-dependent direct cell contact in SSc. In vivo reduction of PD-L1 expression on MSCs confirmed that their antifibrotic effects require PD-L1 binding to PD-1 on T cells. In vitro co-culture experiments further revealed that MSCs specifically inhibit proliferation of PD-1^+^ T cells in SSc-ILD without causing apoptosis. Mechanistically, inflammatory conditions promote PD-L1 upregulation on MSCs, arresting T cells at the G0/G1 phase to block cell division.^42^ This identifies a new MSC-mediated immune checkpoint mechanism for direct T-cell regulation, supporting PD-L1/PD-1-targeted therapeutic strategies for SSc-ILD.

This study demonstrates that MSCs alleviate and reverse the fibrotic process in SSc-ILD by suppressing CD4^+^PD-1^+^ T cell proliferation through a PD-L1-dependent mechanism. Future studies should validate these mechanisms in larger SSc-ILD cohorts and explore PD-1 expression as a predictive biomarker for MSC responsiveness.

## Supplementary material

10.1136/rmdopen-2025-006324 online supplemental table 1

10.1136/rmdopen-2025-006324 online supplemental figure 1

## Data Availability

Data are available upon reasonable request.

## References

[1] Denton CP, Khanna D (2017). Systemic sclerosis. Lancet.

[2] Cottin V, Brown KK (2019). Interstitial lung disease associated with systemic sclerosis (SSc-ILD). Respir Res.

[3] Perelas A, Silver RM, Arrossi AV (2020). Systemic sclerosis-associated interstitial lung disease. Lancet Respir Med.

[4] Khanna D, Tashkin DP, Denton CP (2020). Etiology, Risk Factors, and Biomarkers in Systemic Sclerosis with Interstitial Lung Disease. Am J Respir Crit Care Med.

[5] Papazoglou A, Huang M, Bulik M (2022). Epigenetic Regulation of Profibrotic Macrophages in Systemic Sclerosis-Associated Interstitial Lung Disease. Arthritis Rheumatol.

[6] Goldman N, Ong VH, Denton CP (2024). Pathogenesis of interstitial lung disease in systemic sclerosis. Rheumatol Immunol Res.

[7] Cerro Chiang G, Parimon T (2023). Understanding Interstitial Lung Diseases Associated with Connective Tissue Disease (CTD-ILD): Genetics, Cellular Pathophysiology, and Biologic Drivers. Int J Mol Sci.

[8] Distler O, Highland KB, Gahlemann M (2019). Nintedanib for Systemic Sclerosis-Associated Interstitial Lung Disease. N Engl J Med.

[9] Highland KB, Distler O, Kuwana M (2021). Efficacy and safety of nintedanib in patients with systemic sclerosis-associated interstitial lung disease treated with mycophenolate: a subgroup analysis of the SENSCIS trial. Lancet Respir Med.

[10] Raghu G, Montesi SB, Silver RM (2024). Treatment of Systemic Sclerosis-associated Interstitial Lung Disease: Evidence-based Recommendations. An Official American Thoracic Society Clinical Practice Guideline. Am J Respir Crit Care Med.

[11] Zhang Y, Zhao Y, An C (2025). Material-driven immunomodulation and ECM remodeling reverse pulmonary fibrosis by local delivery of stem cell-laden microcapsules. Biomaterials.

[12] Han H, Chen B-T, Liu Y (2025). Engineered Stem Cell Booster Breaks Pathological Barriers to Treat Chronic Pancreatitis. Adv Mater.

[13] Yuan W, Liu M, Yang D (2025). Improvement in long-term survival with mesenchymal stem cell transplantation in systemic sclerosis patients: a propensity score-matched cohort study. Stem Cell Res Ther.

[14] Alip M, Wang D, Zhao S (2024). Umbilical cord mesenchymal stem cells transplantation in patients with systemic sclerosis: a 5-year follow-up study. Clin Rheumatol.

[15] Fang Y-F, Zhang C, Han M-M (2025). Engineered MSCs Break Endothelial-Myofibroblast Crosstalk in Pulmonary Fibrosis: Reconstructing the Vascular Niche. Adv Mater.

[16] Ganesan N, Chang Y-D, Hung S-C (2023). Mesenchymal stem cells suppressed skin and lung inflammation and fibrosis in topoisomerase I-induced systemic sclerosis associated with lung disease mouse model. Cell Tissue Res.

[17] Akiyama K, Chen C, Wang D (2012). Mesenchymal-stem-cell-induced immunoregulation involves FAS-ligand-/FAS-mediated T cell apoptosis. Cell Stem Cell.

[18] Elahee M, Mueller AA, Wang R (2024). A PD-1(high)CD4(+) T Cell Population With a Cytotoxic Phenotype is Associated With Interstitial Lung Disease in Systemic Sclerosis. ACR Open Rheumatol.

[19] Celada LJ, Kropski JA, Herazo-Maya JD (2018). PD-1 up-regulation on CD4^+^ T cells promotes pulmonary fibrosis through STAT3-mediated IL-17A and TGF-β1 production. Sci Transl Med.

[20] Tan J, Xue Q, Hu X (2024). Inhibitor of PD-1/PD-L1: a new approach may be beneficial for the treatment of idiopathic pulmonary fibrosis. J Transl Med.

[21] Chen Q, Ao L, Zhao Q (2025). WTAP/YTHDF1-mediated m^6^A modification amplifies IFN-γ-induced immunosuppressive properties of human MSCs. J Adv Res.

[22] van den Hoogen F, Khanna D, Fransen J (2013). 2013 classification criteria for systemic sclerosis: an American college of rheumatology/European league against rheumatism collaborative initiative. Ann Rheum Dis.

[23] Hoffmann-Vold A-M, Maher TM, Philpot EE (2020). The identification and management of interstitial lung disease in systemic sclerosis: evidence-based European consensus statements. Lancet Rheumatol.

[24] Hübner R-H, Gitter W, El Mokhtari NE (2008). Standardized quantification of pulmonary fibrosis in histological samples. Biotechniques.

[25] Gao Y, Chi Y, Chen Y (2023). Multi-omics analysis of human mesenchymal stem cells shows cell aging that alters immunomodulatory activity through the downregulation of PD-L1. Nat Commun.

[26] Qin L, Lin H, Zhu F (2023). CD4+LAG3+T cells are decreased in SSc-ILD and affect fibroblast mesenchymal transition by TGF-β3. iScience.

[27] Mo C, Zeng Z, Deng Q (2018). Imbalance between T helper 17 and regulatory T cell subsets plays a significant role in the pathogenesis of systemic sclerosis. Biomed Pharmacother.

[28] Jiang N, Li M, Zeng X (2014). regulatory T cells with clinical parameters in patients with systemic sclerosis. Chin Med J (Engl).

[29] Kuzumi A, Yoshizaki A, Matsuda KM (2021). Interleukin-31 promotes fibrosis and T helper 2 polarization in systemic sclerosis. Nat Commun.

[30] Radstake TRDJ, van Bon L, Broen J (2009). Increased frequency and compromised function of T regulatory cells in systemic sclerosis (SSc) is related to a diminished CD69 and TGFbeta expression. PLoS ONE.

[31] Padilla CM, Valenzi E, Tabib T (2024). Increased CD8+ tissue resident memory T cells, regulatory T cells and activated natural killer cells in systemic sclerosis lungs. Rheumatology (Oxford).

[32] Moog MT, Hinze C, Bormann T (2022). B Cells Are Not Involved in the Regulation of Adenoviral TGF-β1- or Bleomycin-Induced Lung Fibrosis in Mice. J Immunol.

[33] Harada S, Kato M, Nakagome K (2025). Evaluating the Diagnostic Value of Lymphocyte Subsets in Bronchoalveolar Lavage Fluid and Peripheral Blood Across Various Diffuse Interstitial Lung Disease Subtypes. Biomolecules.

[34] Zhao Y, Hao C, Li M (2022). PD-1/PD-L1 inhibitor ameliorates silica-induced pulmonary fibrosis by maintaining systemic immune homeostasis. Biomed Pharmacother.

[35] Maria ATJ, Toupet K, Maumus M (2016). Human adipose mesenchymal stem cells as potent anti-fibrosis therapy for systemic sclerosis. J Autoimmun.

[36] Rozier P, Maria A, Goulabchand R (2018). Mesenchymal Stem Cells in Systemic Sclerosis: Allogenic or Autologous Approaches for Therapeutic Use?. Front Immunol.

[37] Assar S, Mohammadzadeh D, Norooznezhad AH (2023). Improvement in the clinical manifestations of interstitial lung disease following treatment with placental mesenchymal stromal cell extracellular vesicles in a patient with systemic sclerosis: A case report. Respir Med Case Rep.

[38] Liu M, Zeng X, Wang J (2016). Immunomodulation by mesenchymal stem cells in treating human autoimmune disease-associated lung fibrosis. Stem Cell Res Ther.

[39] Liu M, Ren D, Wu D (2015). Stem Cell and Idiopathic Pulmonary Fibrosis: Mechanisms and Treatment. Curr Stem Cell Res Ther.

[40] Toonkel RL, Hare JM, Matthay MA (2013). Mesenchymal stem cells and idiopathic pulmonary fibrosis. Potential for clinical testing. Am J Respir Crit Care Med.

[41] Kletukhina S, Mutallapova G, Titova A (2022). Role of Mesenchymal Stem Cells and Extracellular Vesicles in Idiopathic Pulmonary Fibrosis. Int J Mol Sci.

[42] Li H, Zheng C, Han J (2021). PD-1/PD-L1 Axis as a Potential Therapeutic Target for Multiple Sclerosis: A T Cell Perspective. Front Cell Neurosci.

