# Supplementary figures and images for: Mesenchymal stromal cells ameliorate systemic sclerosis-interstitial lung disease via PD-1/PD-L1 signalling axis
Source: RMD Open. 2026 Jan 6;12(1):e006324. doi: 10.1136/rmdopen-2025-006324 (PMC12778334; doi:10.1136/rmdopen-2025-006324)

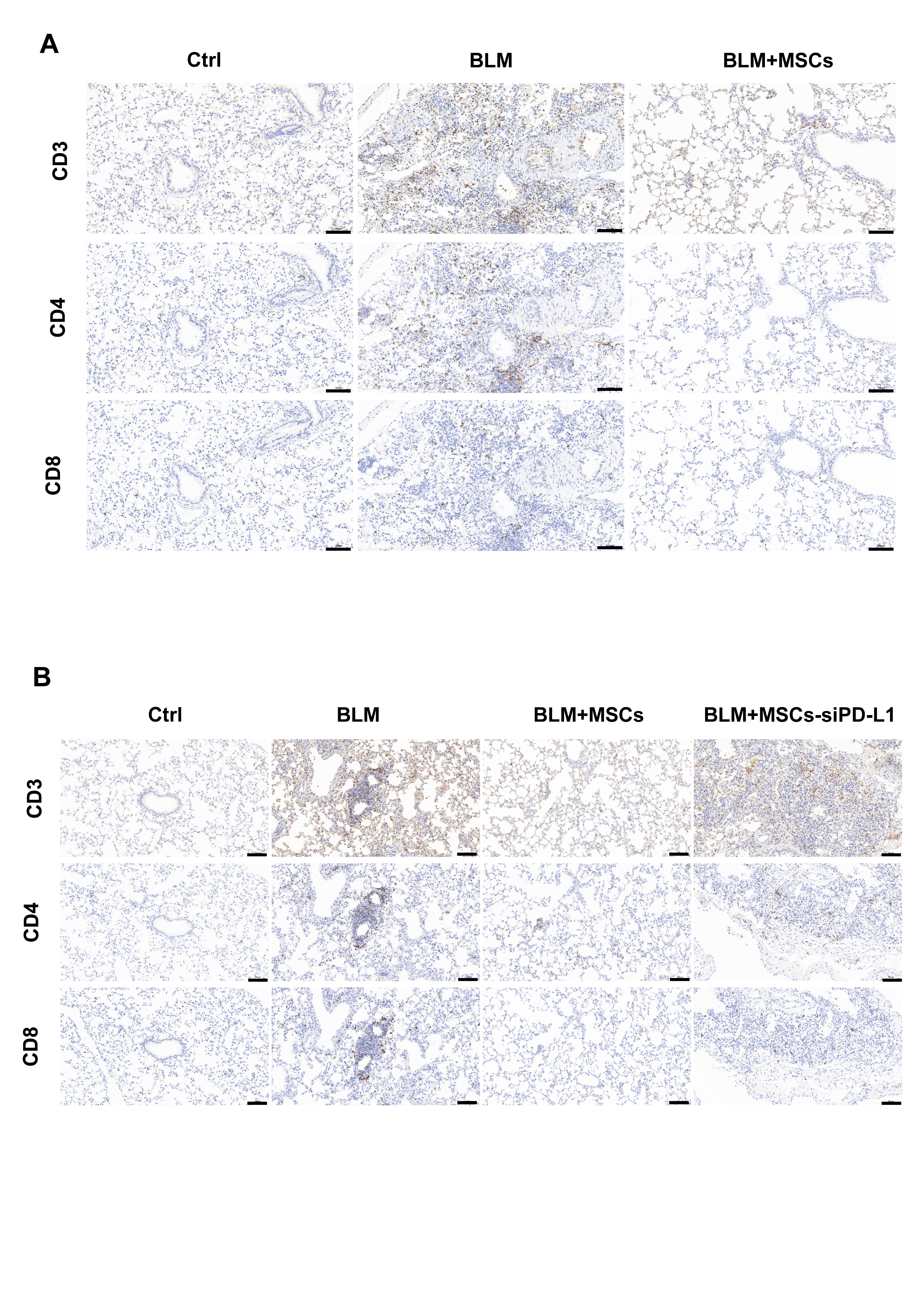

Supplement: online supplemental figure 1 [file rmdopen-12-1-s002.tif]
